# A Novel Itera-Like Densovirus Isolated by Viral Metagenomics from the Sea Barley *Hordeum marinum*

**DOI:** 10.1128/genomeA.01196-14

**Published:** 2014-12-04

**Authors:** S. François, P. Bernardo, D. Filloux, P. Roumagnac, N. Yaverkovski, R. Froissart, M. Ogliastro

**Affiliations:** a INRA, UMR 1333, DGIMI, Montpellier, France; b INRA-CIRAD-SupAgro, UMR 385, BGPI, Campus International de Baillarguet, Montpellier, France; c Service Écologie Végétale, Fondation Tour du Valat, Le Sambuc, Montpellier, France; d CNRS-IRD-UM1-UM2, UMR 5290, MIVEGEC, Montpellier, France

## Abstract

Densoviruses (DVs) infect arthropods and belong to the *Parvoviridae* family. Here, we report the complete coding sequence of a novel DV isolated from the plant *Hordeum marinum* (*Poaceae*) by viral metagenomics, and we confirmed reamplification by PCR. Phylogenetic analyses showed that this novel DV is related to the genus *Iteradensovirus*.

## GENOME ANNOUNCEMENT

Densoviruses (DVs) are small nonenveloped icosahedral viruses infecting arthropods, including pests and vectors for which they are considered biocontrol agents. They contain a single-strand linear DNA genome ranging from 4 to 6 kb, ended by inverted terminal repeats (ITRs) (1). Only 15 DV species are referenced in GenBank so far (2); they display a large diversity of sequences, structures, and organizations. Such diversity, together with the diversity of their invertebrate hosts, suggests that DVs are largely unknown and ubiquitous in the environment. It is crucial to understand the densovirus diversity and prevalence for both fundamental and applied virology issues.

A novel densovirus was detected from sea barley (*Hordeum marinum*) using a virion-associated nucleic acid (VANA) viral metagenomic approach (3). To complement this genome, we performed Rapid Amplification of cDNA Ends (RACE) (Roche), and the products were cloned in the pGEM-T Easy Vector (Promega) and sequenced. The sequences were assembled using Geneious 7.1.4 and compared to database sequences using BLASTn, BLASTp, and tBLASTx (4). The results were considered to be indicative of significant homology when BLAST *E* values were <10^−3^. The genome of this novel DV consists of 4,734 nucleotides (nt), with short ITRs of 130 and 77 nucleotides (nt) at the 3′ and 5′ ends, respectively. The iteravirus genome size is about 5 kb, with ITRs of 250 nt, suggesting that the ITRs of this novel densovirus are not complete (5). The genomic organization of this densovirus is monosense, with three predicted intronless open reading frames (ORFs) encoding two nonstructural proteins (NS) and one structural protein (VP). ORF1 (nt 253 to 2505) has a coding capacity of 750 amino acids (aa) and contains the typical nonstructural 1 (NS1) helicase superfamily III. ORF2 (nt 2559 to 4568) encodes a 669-aa protein corresponding to VP, and it contains the characteristic phospholipase A2 motif (6). ORF3 (nt 380 to 1729) has a coding capacity for NS2 of 449 aa and typically overlapped NS1. The alignment of the VP and NS protein sequences using Clustal W 1.8.1 (7) revealed that this genome had the highest identity (84.9%) with *Danaus plexippus plexippus* densovirus (DpplDV) (GenBank accession no. KF963252) (8). This genome was independently purified from leaves of the original plant stored at −80°C (Qiagen plant DNeasy kit). The PCR products were obtained from different leaves using 15 pairs of primers covering the whole genome that were sequenced using Sanger’s method (Cogenics). Recombination analyses using RDP4.18 (9) revealed that this novel DV might result from an intragenus recombination event between DpplDV and *Dendrolimus punctatus* densovirus (DpDV).

No insect has been found in any part of this plant, no reads obtained from this plant were assigned to arthropods, and no products were obtained using an insect DNA bar coding based on the PCR amplification of a fragment of the mitochondrial cytochrome *c* oxidase subunit I gene (10). This densovirus might come from contamination of the plant aerial part by infected arthropods or circulate systemically *in planta*, as already reported (11). This virus was tentatively named *H. marinum* densovirus (HormaDV).

### Nucleotide sequence accession number.

The GenBank accession no. of HormaDV is KM576800.
